# Machine Learning‐Based Depression Recognition With Preserved Efficacy From Compressed EEG Signals Using Wavelet Transform and Adaptive Filtering

**DOI:** 10.1049/htl2.70084

**Published:** 2026-05-10

**Authors:** Marjan Rezakhani Taleghani, Hadi Grailu

**Affiliations:** ^1^ Electrical Engineering Department Shahrood University of Technology Shahrood Iran

**Keywords:** adaptive filter, compression, diagnosis of depression disorder, electroencephalogram (EEG), machine learning, wavelet transform

## Abstract

Electroencephalogram (EEG) signals are critical for diagnosing neurological disorders like depression but require substantial storage, posing challenges for telemedicine. This paper proposes a novel lossy compression method integrating two‐dimensional zigzag and spiral rearrangement, wavelet transform (bior4.4), and adaptive filtering to minimise storage while preserving depression recognition accuracy. Using the Hospital University Sains Malaysia (HUSM) dataset (34 depressed, 30 healthy subjects), EEG signals were converted into 2D matrices, compressed with four wavelet encoders (SPIHT, STW, EZW, LVL‐MMC), and reconstructed. A feedforward artificial neural network (ANN) with 80 features (relative wavelet energy and entropy) classified depressed and healthy individuals. The combination of zigzag and STW methods achieved a compression ratio (CR) of 89.30, a percentage root‐mean‐square distortion (PRD) of 0.23, and a peak signal‐to‐noise ratio (PSNR) of 58.80 dB, with depression recognition accuracy of 90.2%. Classification was performed on both original and reconstructed signals to evaluate the effect of compression on diagnostic performance. Notably, compressed signals occasionally outperformed original signals, likely due to noise reduction. This method offers a robust solution for efficient EEG storage and accurate depression diagnosis in resource‐constrained settings.

## Graphical abstract

I have discussed various compression methods on electroencephalography signals (EEG) (a biomedical signal extracted from the brain) to emphasise that the compression method chosen should eliminate redundant information while preserving clinically relevant data within the EEG signal. I have proposed a novel approach for compressing and reconstructing EEG signals using a combination of wavelet transform and adaptive filters, with a particular focus on maintaining optimal depression recognition performance. I introduce two array arrangements, namely Zigzag and Spiral, to convert the input EEG signal into a two‐dimensional format. Additionally, I explore four different wavelet encoders for compressing the encoded signal. To assess the compression performance of our proposed method, I employ feedforward artificial neural networks for depression recognition.

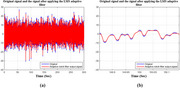

## 1 Introduction

Depression, affecting over 280 million people globally [1], necessitates accessible diagnostic tools. It is also expected to become the second cause of death worldwide, after heart disease [2]. Moreover, traditional diagnostic methods relying on interviews and questionnaires are time‐consuming and resource‐intensive [3]. Electroencephalogram (EEG) signals, which are non‐invasive [4] non‐stationary, non‐linear, and complex characteristics [5], are vital for detecting neurological disorders [6]. However, its challenges include the need for significant storage due to long‐term recordings, multiple channels, and high sampling rates [7, 8]. In telemedicine, where storage is limited, EEG compression is essential [9]. Lossy compression methods, while achieving high compression ratios (CR), risk losing clinically relevant information, potentially compromising diagnostic accuracy [10, 11].

Understanding how depression alters EEG characteristics is therefore essential when evaluating signal processing techniques such as compression. Recent studies have consistently demonstrated that depression is associated with alterations in EEG signals across multiple dimensions. Specifically, changes in spectral power and reduced neural plasticity have been reported, with depressed individuals often failing to exhibit the typical task‐related increases in beta activity [12]. Quantitative EEG studies have also shown differences in absolute and relative spectral power between individuals with major depressive disorder and healthy controls, suggesting alterations in brain function. In particular, changes in theta and alpha power, as well as asymmetry in theta, alpha, and beta bands, have been proposed as potential neurophysiological markers of depression [13, 14]. Previous research [15] has analysed several EEG features, including frequency band power, ERP components, and measures of functional and nonlinear connectivity, demonstrating their effectiveness in distinguishing individuals with depression from healthy controls. Among the most well‐established biomarkers is frontal alpha asymmetry, which has been confirmed through meta‐analysis as a reliable indicator of depression [16]. Moreover, depression is associated with disrupted functional connectivity patterns, reflecting impaired communication between brain regions involved in emotional and cognitive processing [17]. These findings indicate that depression leads to measurable neurophysiological changes in brain activity, making EEG a suitable and objective tool for distinguishing depressed individuals from healthy subjects.

Existing EEG compression techniques include Discrete Cosine Transform (DCT) [8], Multilayer Perceptron (MLP) [18], Set Partitioning in Hierarchical Trees (SPIHT) [19, 20], and DWT‐based ones [21].

These methods offer different balances between compression efficiency and signal quality. Despite significant advances in EEG‑based depression detection, most existing studies do not take data storage and transmission constraints into account, particularly in telemedicine and remote monitoring environments. Furthermore, the effect of lossy EEG compression on diagnostic accuracy remains largely unexplored. Although recent EEG‑based depression recognition approaches such as those using non‑linear features [22] and wavelet‑based techniques [23] have achieved high accuracies, none of these works applied any form of compression.

This study proposes a novel lossy compression method combining two‐dimensional Zigzag and Spiral rearrangement, wavelet transform (bior4.4), and adaptive filtering to maximise CR while preserving depression recognition accuracy. Our method converts 1D EEG signals into 2D matrices, applies wavelet‐based compression, and reconstructs signals for depression classification using an ANN with relative wavelet energy (RWE) and entropy features. Evaluated on the HUSM dataset, the method achieves a CR of 89.30, PRD of 0.23, and PSNR of 58.80 dB, with up to 90% classification accuracy. This work demonstrates that lossy compression can enhance depression recognition by reducing noise.

## 2 Materials and Methods

### 2.1 Previous Methods

EEG compression methods are categorised as lossless or lossy. Lossless methods, such as Huffman coding [8] and arithmetic coding [19], preserve all signal information but achieve low CRs [8]. Lossy methods, including DCT [7], SPIHT [19], and Discrete Wavelet Transform (DWT)‐based approaches [15], offer higher CRs but may compromise clinical data. In [21], a 2D Discrete Wavelet Transform (2D‐DWT) with Haar wavelet lifting and an optimised SPIHT algorithm was proposed for efficient resource utilisation. The scheme in [24] achieves lossy to near‐lossless compression through a two‐stage process, signal normalisation followed by coding with an Integer Fraction Coder (IFC), comprising a spatial coder and a pseudo coder. In [18], a near‐lossless compression method combines DCT with an adaptive ANN‐based transform to reduce EEG data dimensionality, followed by quantisation of the difference between original and estimated DCT coefficients and then arithmetic coding of the quantised error. In [19], EEG signals are organised into a matrix using a two‐stage process before compression. The first stage employs SPIHT for lossy coding, followed by arithmetic coding to encode residuals in the second stage. Other approaches, such as autoencoders [25] and compressive sensing [26], are examples of other techniques applied to EEG signal compression.

For depression recognition, machine learning techniques, such as non‐linear features [22] and convolutional neural networks (CNNs) [27], achieve high classification accuracies. Wavelet‐based methods, as in [23], leverage sub‐band analysis for EEG signal classification. In [28], power spectral density and activity features are extracted using auto‐regression models and Hjorth algorithms with varied time windows. These features are processed via two approaches: ensemble learning, where a deep forest transforms features for enhanced engineering followed by Support Vector Machine (SVM) classification, and deep learning, where a CNN incorporates spatial information from EEG caps through image conversion. Similarly, [29] proposes a three‐channel orthogonal wavelet filter bank (TCOWFB) for depression detection, decomposing EEG signals into seven wavelet sub‐bands (WSBs) using an optimal six‐length TCOWFB. The logarithm of the L2 norm is computed for six detailed and one approximate WSB, serving as discriminating features for a Least Squares Support Vector Machine (LS‐SVM) classifier. In [30], a multimodal model fuses EEG data from neutral, negative, and positive audio stimuli using feature‐level fusion and linear combination techniques. Linear and nonlinear features are extracted, weighted by genetic algorithms, and classified using *k*‐nearest neighbour (KNN), decision tree (DT), and SVM classifiers. However, integrating compression and depression recognition remains underexplored.

### 2.2 Proposed Method

#### 2.2.1 Dataset

The HUSM dataset includes 34 depressed patients (17 men and 17 women, mean age 40.3 ± 12.9 years) and 30 healthy controls (21 men and 9 women, mean age 38.3 ± 15.6 years), approved by the HUSM ethics committee. EEG signals were recorded at a sampling rate of 250 Hz in 5 min open‐eye (EO), 5 min closed‐eye (EC) and 10 min ERP using a three‑stimulus visual oddball paradigm. EEG acquisition was performed using a 19‑channel electro‑gel cap based on the international 10–20 system [23].

A total of 192 EEG recordings (3 per participant) were initially collected. After artefact removal and quality assessment, 18 sessions were excluded due to signal corruption or missing data. The final dataset consisted of 174 valid EEG samples used for training and evaluation. In this study, four channels (FP1, FP2, T3, and T4) were used for analysis due to their relevance to frontal and temporal brain activity associated with major depressive disorder.

#### 2.2.2 Overview

**FIGURE 1:**
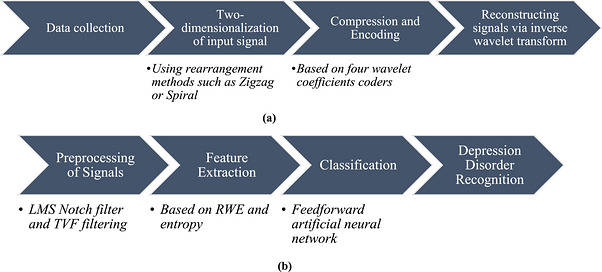
(a) Block diagram of the proposed EEG compression method using Zigzag/Spiral rearrangement, wavelet transform (bior4.4), and EZW, SPIHT, STW, and LVL‐MMC encoding. (b) Depression recognition pipeline using ANN with relative wavelet energy and entropy features.

**FIGURE 2:**
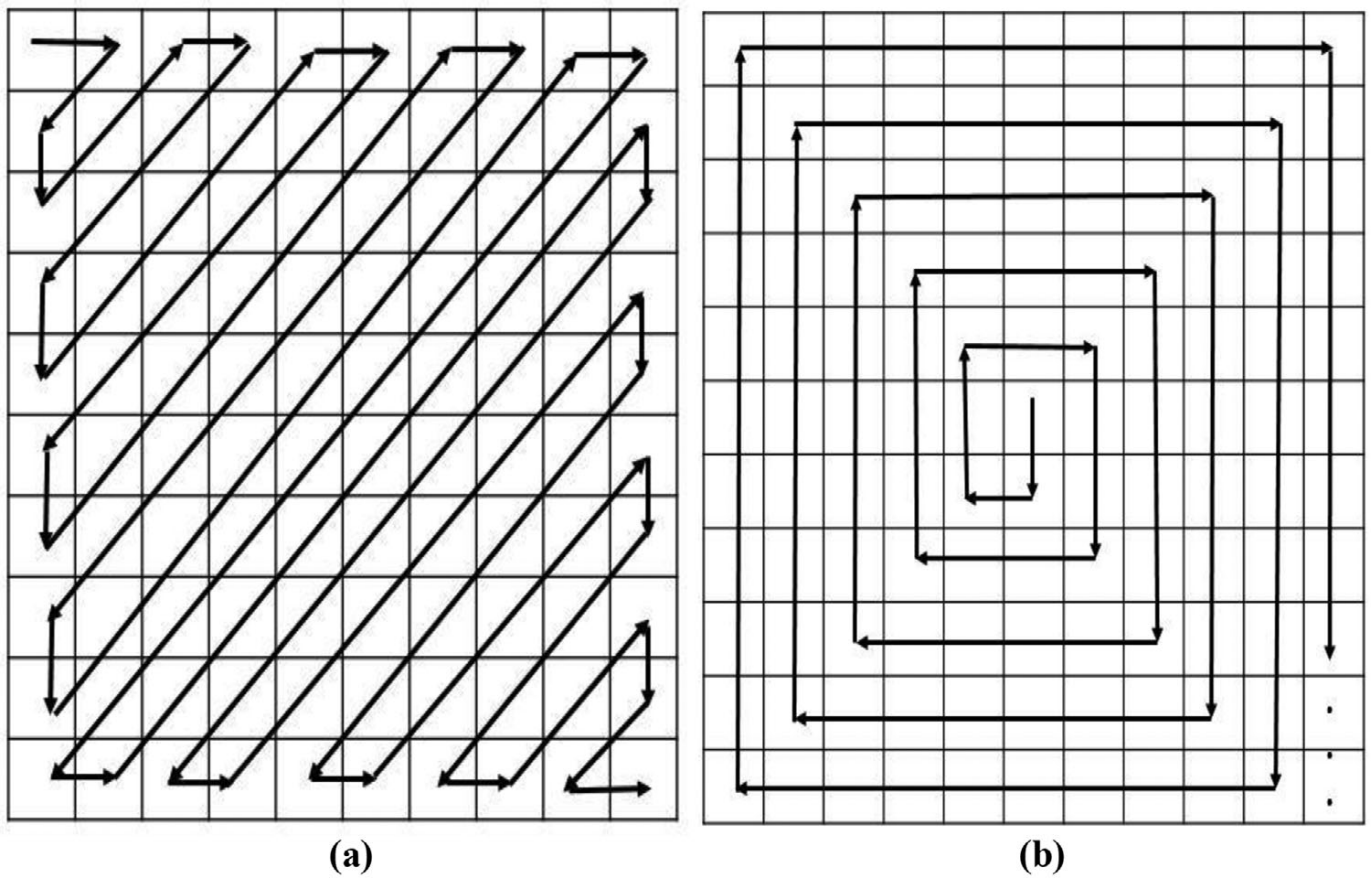
Sample EEG signal rearranged using (a) Zigzag and (b) Spiral methods.

The proposed method compresses EEG signals while preserving diagnostic information for depression recognition. As shown in Figure 1a, the process of compression signals includes (1) converting 1D EEG signals into 2D matrices using Zigzag or Spiral rearrangement (depicted in Figure 2), (2) applying wavelet transform (bior4.4) and encoding with four codecs (EZW, SPIHT, STW, and LVL‐MMC), and (3) reconstructing signals via inverse wavelet transform. Depression recognition, depicted in Figure 1b, uses an ANN with 80 features extracted from four EEG channels (FP1, FP2, T3, T4). Also, the depression recognition process was applied to the original signals and the reconstructed signals obtained after the compression process.

#### 2.2.3 Signal Rearrangement

EEG signals are rearranged into 2D matrices to exploit spatial and temporal redundancies:

Zigzag: Arranges samples diagonally (Figure 2a), preserving local correlations.
Spiral: Organises samples circularly (Figure 2b), capturing broader dependencies.

Zigzag and Spiral were selected for their compatibility with EEG signal characteristics, achieving higher CR and PSNR.

#### 2.2.4 Compression and Reconstruction

The 2D EEG matrix undergoes wavelet transform using bior4.4, chosen for its orthogonality and smoothness. Comparative tests with Daubechies‐4 and Haar wavelets confirmed bior4.4's superiority. Four encoders (SPIHT, STW, EZW, and LVL‐MMC) compress the wavelet coefficients. STW outperformed others due to efficient coefficient partitioning. Reconstruction reverses the process, converting the bitstream to a 1D EEG signal.

#### 2.2.5 Preprocessing and Feature Extraction

**FIGURE 3:**
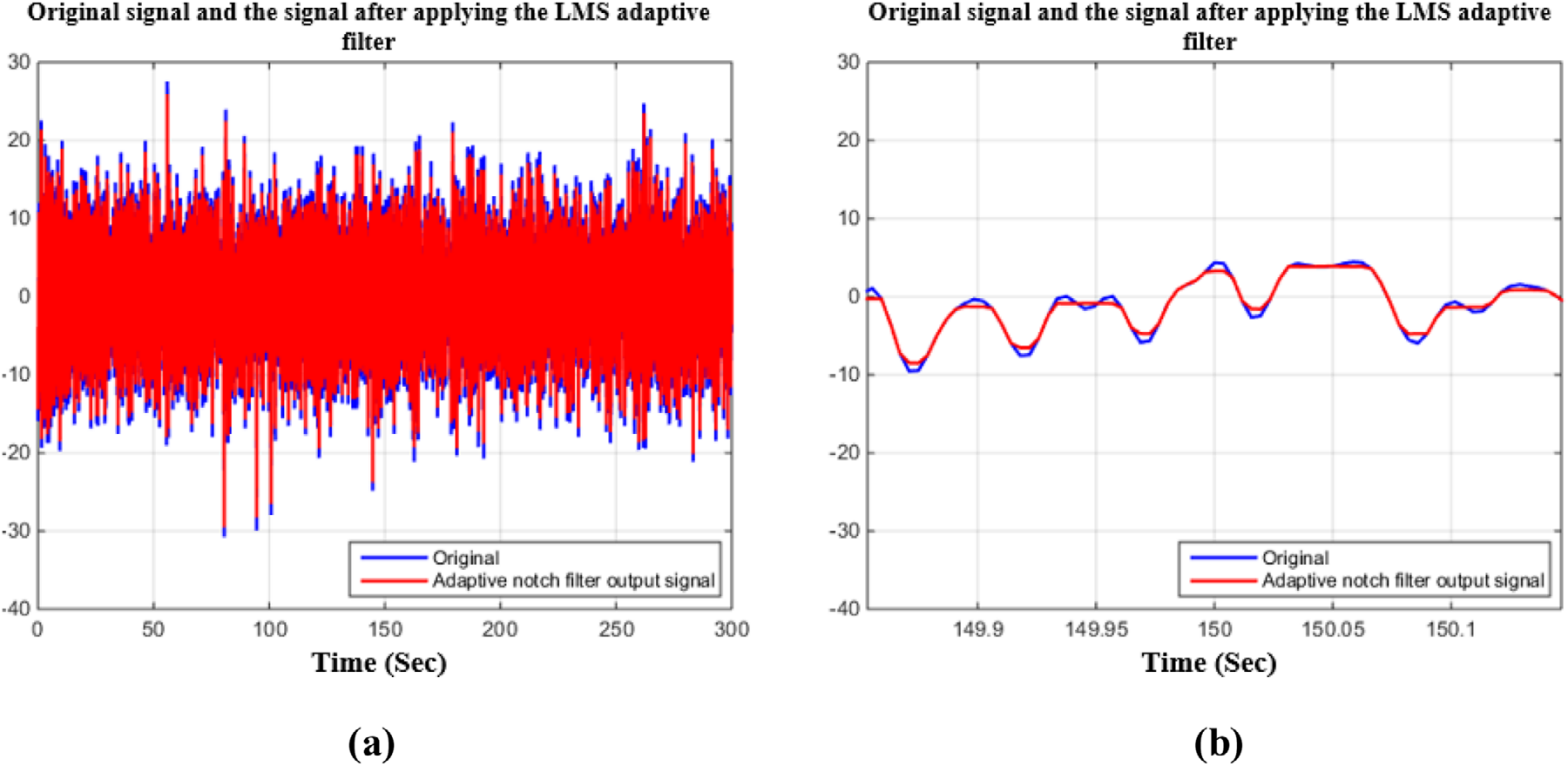
A sample of the original EEG signal from the HUSM dataset before and after applying the LMS Notch filter (step size = 0.1) to remove 50 Hz powerline noise is shown entirely and with an enlarged part (a) and (b).

**FIGURE 4:**
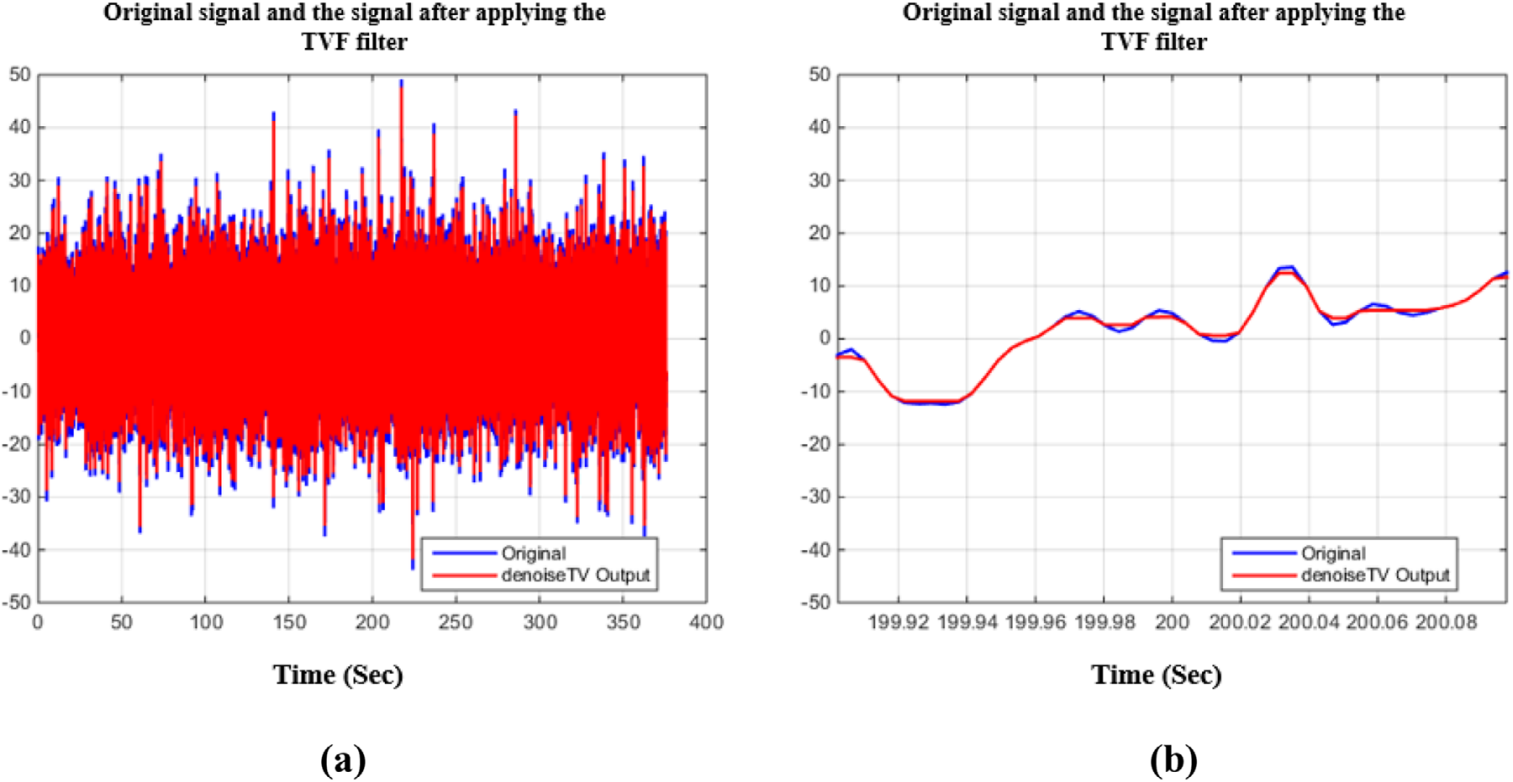
A sample of the original EEG signal from the HUSM dataset before and after applying the TVF denoising using the coiflet‐5 wavelet (λ = 2) is shown entirely and with an enlarged part (a) and (b).

EEG signals are preprocessed to remove noise:

**LMS notch filter**: Eliminates 50 Hz powerline noise with a step size of 0.1, as shown in Figure 3 by a sample EEG signal from the HUSM dataset, where signal peaks are noticeably smoother after filtering.
**Total variation filtering (TVF)**: Removes high‐frequency noise using a coiflet‐5 mother wavelet (λ = 2), chosen for its compatibility with EEG signal characteristics, as demonstrated in Figure 4 with a sample EEG signal from the HUSM dataset, showing reduced peak amplitudes after filtering.

The TVF denoising process estimates the corrupted signal x by minimising the objective function as shown in Equation (1), employing the iterative clipping algorithm [31].

(1) $$J\;\left(x\right)={∥y-x∥}_{2}^{2}+\lambda ∥Ax{∥}_{1}$$

Furthermore, *A* is a matrix with dimensions M × N, and λ is the adjusting parameter that governs the smoothing of the signal. Typically, larger values of λ are chosen for more pronounced noise levels.

Following preprocessing with LMS Notch and TVF filters, EEG signals from the HUSM dataset (FP1, FP2, T3, T4 channels; EO, EC, ERP conditions) are decomposed into frequency sub‐bands using an eight‐level Discrete Wavelet Transform (DWT) with a sampling frequency of 256 Hz (Table 1). The frequency bands, derived using Equation (2), range from 0–0.5 Hz (CA8, Delta) to 64–128 Hz (CD1, Gamma).

(2) $$f=\frac{2^{n-m}f_{s}}{2^{n}}\;$$

**TABLE 1:** The frequency bands correspond to the decomposition levels (with a sampling frequency of 256 Hz).

Decomposition signals	Frequency band (Hz)	Associated band
CD1	64–128	Gamma
CD2	32–64	Gamma
CD3	16–32	Beta
CD4	8–16	Alpha
CD5	4–8	Theta
CD6	2–4	Delta
CD7	1–2	Delta
CD8	0.5–1	Delta
CA8	0–0.5	Delta

In Equation (2), *f* denotes the frequency range corresponding to the decomposition level *m*, *fs* is the sampling frequency, and *n* is the number of data points in the signal.

Twenty features are extracted for depression classification using an ANN. Nine features consist of Relative Wavelet Energy (RWE), which quantifies the degree of similarity between segments of a signal and serves as a useful tool for detecting and analysing specific events in both the time and frequency domains [32], calculated per Equation (5) for eight detail sub‐bands (CD1–CD8) and one approximation sub‐band (CA8). Tables 2 and 3 present the average values of these features for normal and depressed EEG signals from the left and right hemispheres, respectively.

**TABLE 2:** Average of RWE of EEG signal in depressed and control groups in the left hemisphere.

RWE	Healthy	Depressed	Frequency band (Hz)
$\rho_{1}$	0.0063	0.00305	64–128
$\rho_{2}$	0.1106	0.0307	32–64
$\rho_{3}$	0.0747	0.1491	16–32
$\rho_{4}$	0.0974	0.14145	8–16
$\rho_{5}$	0.2482	0.15825	4–8
$\rho_{6}$	0.13445	0.28175	2–4
$\rho_{7}$	0.13845	0.1755	1–2
$\rho_{8}$	0.10565	0.0169	0.5–1
$\rho_{9}$	0.08425	0.0433	0–0.5

**TABLE 3:** Average of RWE of EEG signal in depressed and control groups in the right hemisphere.

RWE	Healthy	Depressed	Frequency band (Hz)
$\rho_{1}$	0.0067	0.00465	64–128
$\rho_{2}$	0.1096	0.02845	32–64
$\rho_{3}$	0.07595	0.13885	16–32
$\rho_{4}$	0.108	0.14045	8–16
$\rho_{5}$	0.1945	0.15525	4–8
$\rho_{6}$	0.1496	0.28675	2–4
$\rho_{7}$	0.162	0.1796	1–2
$\rho_{8}$	0.12365	0.0158	0.5–1
$\rho_{9}$	0.07	0.0502	0–0.5

The energy of wavelet coefficients *d_j_*_,_ *_k_* corresponding to the decomposition level *j* is defined by Equation (3).

(3) $$\;E_{j}=\sum_{k}{\left|d_{j,k}\right|}^{2}\;;\;j=1,\text{…},N$$

According to Equation (4), the total energy of the signal is the sum of its sub‐band energies, encompassing both the N detailed sub‐bands and the approximation sub‐band.

(4) $$\;E_{total}=\sum_{j=1}^{N+1}E_{j\;}$$

Now, the Relative Wavelet Energy (RWE) can be defined as per Equation (5).

(5) $$\;\rho_{j}=\frac{E_{j}}{E_{total}},\;j=1,\text{…},N+1$$

Two additional features, *y_1_* (sum of RWE for Beta, Alpha, and Theta: 4–32 Hz) and *y_2_* (sum of RWE for Delta: 0–4 Hz), are defined in Equations (6) and (7) to emphasise the 0–32 Hz range, where most EEG energy is concentrated.

(6) $$\;y_{1}=\rho_{3}+\rho_{4}+\rho_{5}$$

(7) $$\;y_{2}=\rho_{6}+\rho_{7}+\rho_{8}+\rho_{9}$$

Nine wavelet entropy features, computed per Equation (8), quantify signal complexity for each sub‐band. These features, robust for distinguishing depressed and healthy subjects, enable effective ANN‐based classification.

(8) $$\;ENT_{i}=-\sum_{j=1}^{N}D_{ij}^{2}\log\left(D_{ij}^{2}\right),\;i=1,2,\text{…},l$$

where *i* is the wavelet decomposition level.

#### 2.2.6 Classification

**FIGURE 5:**
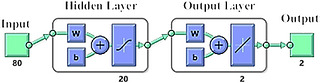
Block diagram of using a neural network.

A feedforward ANN with 20 hidden neurones and a Tansig activation function classifies depressed and healthy subjects. Activation functions are essential for enabling neural networks to model complex patterns for determination [33]. They transform input signals into a suitable output range, facilitating the learning of intricate input‐output relationships [34]. Four EEG channels (FP1, FP2, T3, and T4) are utilised, and the network input consists of 80 features (20 per channel), as shown in Figure 5.

To avoid any data leakage, a subject‐wise cross‐validation procedure was adopted. Specifically, a Leave‐One‐Subject‐Out (LOSO) strategy was implemented, in which all data from one subject were held out exclusively for testing, while the remaining subjects were used for training (and internal validation). This ensures that the model is consistently evaluated on completely unseen subjects, providing a reliable assessment of its generalisation performance.

### 2.3 Evaluation Metrics

Compression performance was evaluated using:

Compression Ratio (CR): Ratio of original to compressed data size (Equation 9).

(9) $$CR=\frac{S_{o}}{S_{c}}$$

where $S_{o}$ and $S_{c}$ represent the original and compressed EEG signals, respectively.

Percentage root‐mean‐square distortion (PRD): Reconstruction error (Equation 10).

(10) $$PRD=\sqrt{\frac{\sum_{i=1}^{L}{\left(x_{i}-\hat{x_{i}}\right)}^{2}}{\sum_{i=1}^{L}{\left(x_{i}\right)}^{2}}}\;\times 100$$

where $x_{i}$ represents the original signal, ${\hat{x}}_{i}$ denotes the reconstructed signal, and *L* is the length of the signal.

Peak Signal‐to‐Noise Ratio (PSNR): PSNR serves as a metric for assessing the similarity between the two‐dimensional image before and after compression, and it is expressed in decibels (dB). PSNR can be defined as shown in Equation (11).

(11) $$\mathrm{PSNR}\;=10\;\times \;log_{10}\left(\frac{Q^{2}}{MSE}\right)$$

where Q, is 255.

Also, MSE is defined as in Equation (12).

(12) $$\mathrm{MSE}\;=\frac{1}{mn}\sum_{i=0}^{m-1}\sum_{j=0}^{n-1}{\left[I\left(i,j\right)-K\left(i,j\right)\right\}}^{2}$$

Depression recognition was assessed using:

Accuracy: Correct classification rate (Equation (13)).
(13) $$\mathrm{Accuracy}\;=\frac{TN+TP}{TN+FP+TP+FN}\;\times 100$$
Sensitivity: True positive rate (Equation (14)).
(14) $$\mathrm{sensitivity}=\frac{TP}{TP+FN}\;\times 100\%$$
Selectivity: Precision (Equation (15)).
(15) $$\mathrm{selectivity}=\frac{TP}{TP+FP}\;\times 100\%$$
Specificity: True negative rate (Equation (16)).
(16) $$\mathrm{specificity}\;=\frac{TN}{TN+FP}\;\times 100\%$$

## 3 Results

### 3.1 Compression Performance

Compression performance was evaluated for Zigzag and Spiral rearrangements. Table 4 shows results for Zigzag:

**TABLE 4:** Compression performance (Zigzag rearrangement).

Wavelet CODEC	CR left hemisphere	CR right hemisphere	PRD left hemisphere	PRD right hemisphere	PSNR left hemisphere	PSNR right hemisphere
EZW	100.38 98.23 97.04	98.05 95.30 94.84	1.11 1.09 1.14	1.13 1.06 1.09	45.48 45.71 45.57	45.69 46.01 45.88
	Average:98.55^a^	Average:96.07	Average:1.11	Average:1.09	Average:45.59	Average:45.86
SPIHT	70.67 69.53 68.77	69.02 67.54 67.37	1.11 1.09 1.14	1.13 1.06 1.09	45.48 45.71 45.57	45.69 46.01 45.88
	Average:69.66	Average:67.98	Average:1.11	Average:1.09	Average:45.59	Average:45.86
STW	91.34 90.36 89.22	89.33 88.15 87.72	0.22 0.23 0.23	0.23 0.23 0.23	58.78 58.77 58.78	58.86 58.80 58.79
	Average:90.31	Average:88.40	Average:0.23	Average:0.23	Average:58.78	Average:58.82
LVL‐ MMC	75.85 77.61 73.64	73.38 75.68 72.76	0.29 0.26 0.30	0.30 0.26 0.29	57.15 57.84 56.74	56.83 57.83 56.89
	Average:75.70	Average:73.94	Average:0.28	Average:0.29	Average:57.24	Average:57.19

^a^ Numbers 1, 2, and 3 represent the eyes‑closed (EC), eyes‑open (EO), and ERP conditions, respectively, and the final value reports the average across these three states.

Table 5 shows results for Spiral: Combination of Zigzag and STW methods outperformed other codecs, achieving the highest CR and PSNR due to efficient elimination of negligible coefficients. Zigzag was slightly superior to Spiral.

**TABLE 5:** Compression performance (Spiral rearrangement).

Wavelet CODEC	CR left hemisphere	CR right hemisphere	PRD left hemisphere	PRD right hemisphere	PSNR left hemisphere	PSNR right hemisphere
EZW	70.97 67.16 70.22	69.33 64.92 68.67	0.97 0.94 0.99	0.98 0.91 0.95	46.63 46.93 46.80	46.85 47.25 47.03
	Average:69.45	Average:67.64	Average:0.97	Average:0.95	Average:46.79	Average:47.04
SPIHT	50.38 47.77 49.83	49.21 46.23 48.77	0.97 0.94 0.99	0.98 0.91 0.95	46.63 46.93 46.80	46.85 47.25 47.03
	Average:49.33	Average:48.07	Average:0.97	Average:0.95	Average:46.79	Average:47.04
STW	69.23 66.75 67.35	67.67 64.84 66.16	0.23 0.22 0.22	0.17 0.22 0.22	58.88 58.87 58.96	58.99 58.94 58.97
	Average:67.78	Average:66.23	Average:0.22	Average:0.21	Average:58.90	Average:58.97
LVL‐ MMC	59.77 57.05 57.96	58.01 55.32 56.90	0.29 0.29 0.29	0.30 0.29 0.29	56.89 56.85 56.85	56.70 56.82 57.03
	Average:58.26	Average:56.74	Average:0.29	Average:0.29	Average:56.87	Average:56.85

### 3.2 Depression Recognition Performance

Recognition performance was assessed for original and compressed signals. Table 6 compares them:

**TABLE 6:** Recognition performance without compression (original signals).

States	Accuracy (%)	Sensitivity (%)	Selectivity (%)	Specificity (%)
EC	83.9	82.8	85.2	85.7
EO	93.1	92.6	93.5	92.6
ERP	96.7	100	94.1	92.2
All states (average)	86.2	89.3	83.8	80.7

Table 7 details compressed signal performance:

**TABLE 7:** Recognition performance on compressed EEG signals (combined three states).

Proposed method	Accuracy (%)	Sensitivity (%)	Selectivity (%)	Specificity (%)
Reconstructed using Zigzag and EZW	91.4	93.6	89.6	88
Reconstructed using Zigzag and SPIHT	90.8	92.4	89.5	88
Reconstructed using Zigzag and STW	90.2	94.6	87	84.3
Reconstructed using Zigzag and LVL‐MMC	91.4	92.5	90.4	89.2
Reconstructed using Spiral and EZW	92	96	88.9	86.7
Reconstructed using Spiral and SPIHT	91.4	93.6	89.6	88
Reconstructed using Spiral and STW	92	96	88.9	86.7
Reconstructed using Spiral and LVL‐MMC	90.2	94.6	87	84.3

The proposed model achieved a classification accuracy of 90.2%. To provide a statistical validation of this result, the 95% confidence interval (CI) was computed for the classification accuracy. Based on 157 correct predictions out of 174 samples, the resulting 95% confidence interval for the accuracy is 85.8%–94.6%. The relatively narrow interval demonstrates that the model performance is stable and not significantly affected by data variations.

The improvement in classification performance observed in the compressed signals can be attributed to the denoising effect of the compression process. In the proposed approach, the wavelet transform combined with Zigzag rearrangement tends to preserve the dominant low‐frequency components of the EEG signals while reducing high‐frequency noise and redundant information. As a result, the compression stage acts as a natural filtering mechanism that improves the signal to noise ratio (SNR) and enhances the separability of the extracted features for the ANN classifier. Consequently, the reconstructed signals can achieve slightly better classification performance than the original signals, as reflected in Tables 6 and 7. Although all compression methods were evaluated in the classification stage, the Zigzag–STW combination is highlighted since it achieved superior performance in the compression results.

**FIGURE 6:**
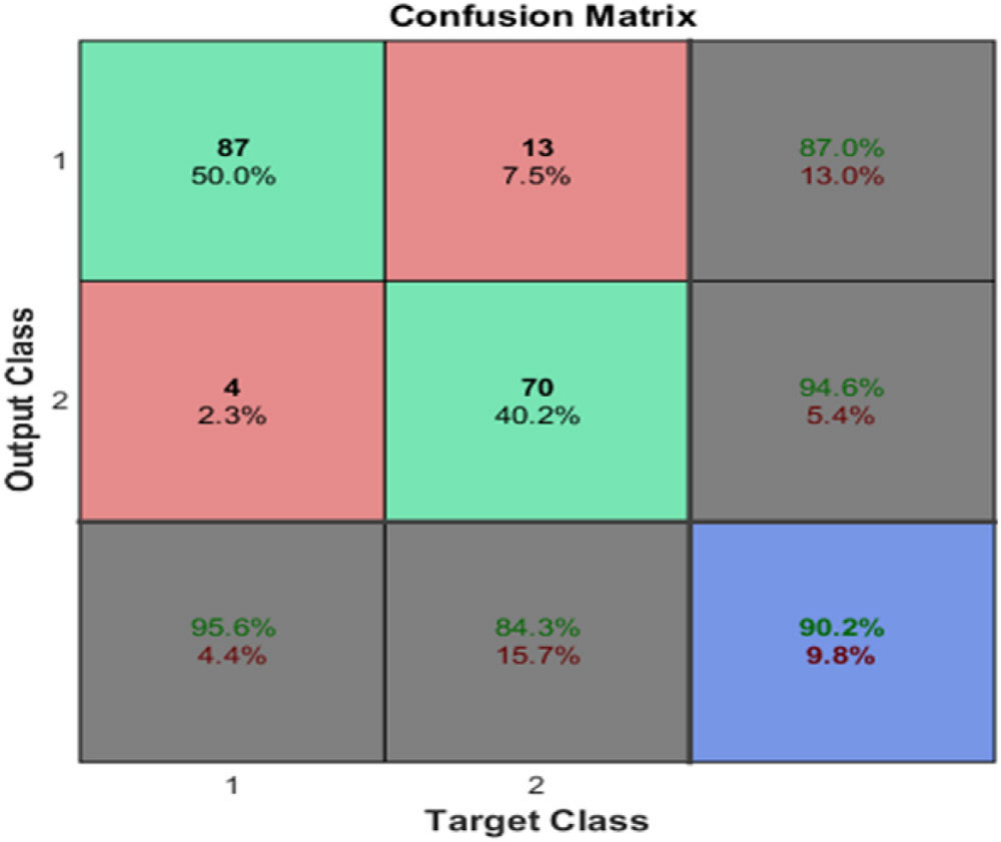
Confusion matrix of the proposed EEG depression classification model.

Furthermore, the confusion matrix corresponding to the classification accuracy of 90.2% is presented in Figure 6.

### 3.3 Comparison With State‐of‐the‐Art

Tables 8 and 9 compare the proposed method with state‐of‐the‐art techniques in terms of EEG compression and depression recognition performance, respectively.

**TABLE 8:** Comparison of compression results.

Method	CR	PRD	PSNR (dB)
DCT and HOFFMAN coding [8]	AVG = 1.78	——	——
DCT Method [7]	4	5.49	——
Combination of DWT and SPIHT [21]	1.95	——	——
Normalised Spatial Pseudo Codec [24]	4.61	5.33	21.42
Combination of DCT and ANN [18]	10.31	5.59	——
Autoencoders [25]			
Proposed method (Zigzag and STW Method)	89.30	0.23	58.80

**TABLE 9:** Depression recognition comparison.

Method	Accuracy (%)	Sensitivity (%)	Specificity (%)
Non‐linear features and LR classifier [22]	90	——	——
Wavelet‐based method [23]	87.5	95	80
CNN [27]	98.32	98.34	——
Deep forest, support vector machine (SVM) and power spectral density [28]	89.02	91.29	86.76
Wavelet‐based method [29]	99.54	98.66	99.38
Information fusion techniques with KNN [30]	86.98	——	——
Proposed method (Zigzag and STW method)	90.2	94.6	84.3

*Note*: Compared to existing methods, the proposed approach achieves competitive accuracy while significantly reducing data size.

## 4 Discussion

The proposed method demonstrates strong compression performance with a compression ratio of 89.30, PRD of 0.23, and PSNR of 58.80 dB. These results indicate that the Zigzag‐STW combination compression framework effectively preserves the clinically relevant components of EEG signals while achieving substantial data reduction.

The depression recognition accuracy of 90.2% further highlights the effectiveness of the overall framework. In addition, the reported accuracy is supported by a 95% confidence interval of 85.8%–94.6%, indicating stable performance across the evaluated samples. In several cases, the reconstructed signals achieved slightly improved classification performance compared with the original signals. This behaviour can be attributed to the denoising effect of the compression process. Specifically, the wavelet transform tends to retain low‑frequency EEG components while reducing high‑frequency noise, resulting in cleaner reconstructed signals that may enhance the ANN classifier's ability to detect relevant patterns.

In terms of computational complexity, the proposed method is efficient due to the use of wavelet transform and signal rearrangement techniques, both of which have relatively low computational overhead. The ANN classifier employed in this study has a shallow architecture, making it computationally lightweight. Moreover, the compression stage significantly reduces the size of EEG data, which directly decreases storage requirements and transmission latency. These characteristics make the proposed method suitable for real‐time applications and telemedicine systems.

Despite the promising results, several limitations should be considered. The HUSM dataset includes 64 subjects, which may limit generalisability, and differences among datasets may affect the fairness of direct comparison.

## 5 Conclusion

This study introduces a novel EEG compression method that preserves depression recognition accuracy. Combining Zigzag or Spiral rearrangement, wavelet transform (bior4.4), and adaptive filtering, the method achieves a CR of 89.30, PRD of 0.23, and PSNR of 58.80 dB, with up to 90% classification accuracy. The improvement in recognition after compression highlights the benefit of noise reduction. Future work will explore larger datasets.

## Author Contributions

Marjan Rezakhani Taleghani contributed to the methodology, coding implementation, analysis, and preparation and writing of the original and revised manuscript. Dr Hadi Grailu provided conceptualisation, supervision and contributed to reviewing and editing the manuscript.

## Funding

The authors have nothing to report.

## Conflicts of Interest

The authors declare no conflicts of interest.

## Data Availability

The data supporting the findings of this study are available at https://figshare.com/articles/EEG_Data_New/4244171.
